# A review of delivery room resuscitation in very low birth weight infants in a middle income country

**DOI:** 10.1186/s40748-017-0048-y

**Published:** 2017-05-30

**Authors:** Daynia E. Ballot, Faustine Agaba, Peter A. Cooper, Victor A. Davies, Tanusha Ramdin, Lea Chirwa, David Rakotsoane, Lethile Madzudzo

**Affiliations:** Department of Paediatrics and Child Health, University of the Witwatersrand, Private Bag X 39, Johannesburg, 2000 South Africa

**Keywords:** Neonate, Resuscitation, Mortality, Delivery room

## Abstract

**Background:**

Advanced levels of delivery room resuscitation in very low birth weight infants are reported to be associated with death and complications of prematurity. In resource limited settings, the need for delivery room resuscitation is often used as a reason to limit care in these infants.

**Methods:**

This was a review of delivery room resuscitation in very low birth weight infants born in a tertiary hospital in South Africa between 01 January 2013 and 30 June 2016. Outcomes included death and serious complications of prematurity. Advanced delivery room resuscitation was defined as the need for intubation, chest compressions or the administration of adrenaline.

**Results:**

A total of 1511 very low birth weight infants were included in the study. The majority (1332/1511 (88.2%) required oxygen in the delivery room. Face mask ventilation was needed in 45.2% (683/1511). Advanced delivery room resuscitation was only required in 10.6% (160/1511). More than half the infants who required advanced delivery room resuscitation died (89/160; 55.6%). Advanced delivery room resuscitation was required in significantly more infants <1000 grams at birth than those infants >1000 grams (83/539 (15.4%) vs 77/972 (7.9%) *p* < 0.001). Advanced delivery room resuscitation was significantly associated with a 5 minute Apgar score < 6 (OR 13.8 (95%CI 8.6–22.0), supplemental oxygen at day 28 (OR 2.2 (95% CI 1.4–3.9), metabolic acidosis (OR 2.3 (95% CI 1.1–4.8) and death (OR 1.9 95% CI 1.1–3.3). Other serious complications of prematurity were not associated with advanced delivery room resuscitation. Mortality was increased in infants with a low admission temperature (35.1 °C (SD 0.92) vs 36.1 °C (SD 1.4) (*p* < 0.001).

**Conclusion:**

There was a high mortality rate associated with advanced delivery room resuscitation; however complications of prematurity were not increased in survivors..The need for advanced delivery room resuscitation alone should not be used as a predictor of poor outcome in very low birth weight infants. Survivors of advanced delivery room resuscitation should be afforded ventilatory support if required. Special care must be taken to avoid hypothermia in very low birth weight infants requiring resuscitation at birth.

## Background

Perinatal asphyxia is an important cause of morbidity and mortality, especially in low and middle income countries. In a review in South Africa in 2007, one third of neonatal deaths were due to asphyxia – hypoxia [1]. In term infants, perinatal asphyxia may result in neonatal encephalopathy, with possible long term neurological sequelae such as tetraplegic cerebral palsy [2]. In a review from the United States, 94% of neonates with an Apgar score of zero at 10 minutes either died or had severe handicap [3]. Therapeutic hypothermia has been shown to reduce rates of both mortality and long term handicap in term infants with moderate to severe hypoxic ischaemic encephalopathy [4].

Hypoxic ischaemic brain injury in preterm infants is less well defined than in their term counterparts; the extent and type of brain damage depends on brain immaturity, cellular vulnerability and the timing and intensity of the asphyxial insult [5]. Advanced delivery room resuscitation (ADRR), defined as chest compressions, intubation or the administration of adrenaline, is significantly more likely in infants of low birth weight and gestational age [6–10]. There is a significantly increased risk of death with ADRR in VLBWI [7–11]. Serious complications of prematurity, including severe intraventricular haemorrhage (IVH), retinopathy of prematurity (ROP), respiratory distress syndrome (RDS), pneumothorax, late onset sepsis (LOS), pulmonary haemorrhage, bronchopulmonary dysplasia (BPD) and use of postnatal steroids, are more common in VLBWI who had ADRR [6–9].

South Africa is a middle income country with limited health funding and resources. There are insufficient neonatal ventilator facilities to provide mechanical ventilation to all those VLBWI who require it. In order to address this problem, mechanical ventilation was rationed to those VLBWI above 1000 grams at birth, based on the premise that extremely low birth weight infants (ELBWI) with a birth weight below 1000 grams have a poor outcome. More recently, however, there has been a move away from using a birth weight cut off alone for mechanical ventilation in VLBWI [12]. Although many VLBWI are now successfully treated with surfactant replacement therapy (SRT) and nasal continuous positive airway pressure (NCPAP) [13], the issue of limited resources for ventilatory support of VLBWI in the South African context remains an important health challenge. The need for ADRR has been suggested as a possible criterion for selecting VLBWI for ventilatory support.

Resuscitation at birth has been reported to be associated with mortality in VLBWI in South Africa [14]. There is, however, limited information on survival and complications of prematurity at different levels of delivery room resuscitation (DRR) in VLBWI in sub-Saharan Africa. The aim of this study is to review DRR in VLBWI, in a tertiary referral centre in South Africa. Primary study objectives were to determine the outcome of DRR and ADRR in VLBWI and to establish whether the need for ADRR should be used as a reason to limit care to VLBWI in resource limited settings. A secondary study objective was to evaluate the association of low admission temperature in VLBWI with the need for ADRR as well as death.

## Methods

This study was conducted in the neonatal unit in a tertiary referral centre, between 01 January 2013 and 30 June 2016. All inborn VLBWI were included in the study. Infants with no record of resuscitation and those with lethal congenital abnormalities were excluded.

Delivery room resuscitation was classified as none, oxygen only, positive pressure ventilation, endotracheal intubation, chest compressions and administration of adrenaline. “Oxygen only” referred to blow –by oxygen. If the infant did not respond to supplemental oxygen alone, oxygen would then be provided using positive pressure ventilation with a T piece resuscitator. An air oxygen blender was not available in the delivery room during the time of the study. A baby could have had all levels of intervention and was analysed accordingly. All VLBWI were wrapped in plastic wrap and resuscitated under a radiant warmer. The delivery room temperature was set at 25 °C.

### Study site

The study site was a tertiary referral centre for both high risk obstetric and neonatal cases. The neonatal unit comprised two areas – a neonatal intensive care unit (NICU) where mechanical ventilation was provided and a premature nursery where VLBWI were treated with surfactant and NCPAP. There were 32081 live births during the study period and the overall Caesarean section (C section) rate was 48.1%. Uncomplicated preterm labour alone was not an indication for C section. Elective C section referred planned delivery by C section e.g. for previous C section and malposition. Spinal anesthesia was routinely used during the study period. Primary indication for C section was recorded.

Preterm deliveries were attended by an intern who was supervised by a registrar. An intern was a newly qualified doctor doing compulsory training in the paediatric unit, as part of a general practical training. A registrar was a doctor specializing in paediatrics. All registrars were completing a 4 year rotation in paediatrics and all had at least 18 months of paediatric experience prior to commencing the registrar rotation. Both registrars and interns were trained in neonatal resuscitation. The intern would initiate neonatal resuscitation and call the registrar for assistance as required. Resuscitation was initiated in all VLBWI irrespective of birth weight, unless there was a known diagnosis of intrauterine fetal death. Resuscitation could be discontinued after 10 minutes of asystole, in consultation with a senior member of staff.

All VLBWI were admitted to a transitional delivery ward nursery for initial evaluation and stabilization; VLBWI were then transferred to the premature nursery or NICU as required. The duration of stay in the transitional nursery could last several hours before a bed in NICU or the premature nursery became available. Deaths that occurred in this transitional nursery or labour room were classified as delivery room deaths. Deaths that occurred within 12 hours of life included VLBWI who died in the delivery room, premature nursery or NICU. Due to limited resources (both staff and equipment), during the study period, surfactant and NCPAP were only provided to VLBWI weighing >750 grams at birth; mechanical ventilation was only provided to VLBWI > 900 grams at birth. A second dose of surfactant could be given if respiratory distress worsened in VLBWI <900 grams. There was no NCPAP or surfactant replacement therapy (SRT) available in the delivery room for resuscitation during the study period. The initial therapy for all VLBWI with respiratory distress syndrome (RDS) was SRT and NCPAP. The axillary temperature of all VLBWI was recorded on admission to the transitional nursery.

### Neonatal resuscitation training

All staff was trained in neonatal resuscitation according to the South African Neonatal Resuscitation Algorithm (SANRA) approved by the South African Resuscitation Council (www. resus.co.za) and the South African Paediatric Association. The SANRA is a single standardised protocol used for training all birth attendants – both nursing and medical. The SANRA states that intubation can be done at any point with a skilled operator, but most birth attendants in South Africa are midwives or junior doctors who are not skilled in endotracheal intubation. This protocol therefore emphasizes the need for proper face mask ventilation and de-emphasizes the need for intubation. Chest compressions were initiated prior to intubation and routine intubation for VLBWI was not recommended.

### Database

This was a secondary analysis of an existing neonatal database, managed using Research Electronic Data Capture (REDCAP) software [15], hosted by the University of the Witwatersrand. Information was collected for each VLBWI upon hospital discharge and entered into the database, which is kept for the purpose of clinical audit. Data was verified against the hospital records after entry into the database. Data analysed for the purpose of the study included maternal information, delivery room details, VLBWI demographics, clinical characteristics and outcome.

### Data analysis

Data was analysed using IBM SPSS version 23. Continuous data was described using mean and standard deviation or median and range, as appropriate, according to the distribution of the data. Categorical variables were described using frequencies and percentages. Outcomes of interest were in hospital mortality or significant complications of prematurity including necrotising enterocolitis (NEC), ROP, IVH, supplemental oxygen at 28 days of life, postnatal steroids, RDS, SRT, NCPAP, mechanical ventilation, patent ductus arteriosus (PDA), neonatal sepsis, cystic periventricular leukomalacia (PVL). Diagnosis of PDA was confirmed on echocardiography by a cardiologist; NEC was defined as modified Bell stage 2 or 3 [16] and IVH as grade 3 or 4 according to the grading of Papile [17]. Sepsis was defined as culture proven sepsis (including coagulase negative staphylococcus) - early onset sepsis (EOS) occurred before 3 days of life, while LOS occurred after 3 days. In-hospital mortality included all deaths from any cause during the hospital stay (not only deaths from perinatal asphyxia). Metabolic acidosis referred to a base excess above-16 mmol/l at any time – not only in the perinatal period. In hospital mortality was compared between different levels of DRR. The level of resuscitation and mortality was compared between ELBWI and those infants > 1000 grams birth weight. Advanced delivery room resuscitation was defined as the need for endotracheal intubation, chest compressions or administration of adrenaline. Outcomes were compared between those VLBWI who were given ADRR and those who had no ADRR. Continuous variables were compared using unpaired t tests or Mann Whitney U as appropriate. Categorical variables were compared using Chi squared tests. A p value <0.05 was considered to be statistically significant. In all analysis, only valid cases were reported (i.e. missing data was not included). Variables which were found to have a statistically significant association with ADRR on univariate analysis were entered into a multivariate logistic regression model.

## Results

### Total sample

There were 2994 VLBWI admitted over the study period. There were 31 infants with congenital abnormalities, 11 infants had no recorded resuscitation, 1430 infants were born elsewhere and 11 infants had no recorded birth weight. The final sample therefore comprised 1511 VLBWI. The mean birthweight of VLBWI was1091.8 (SD260) grams and gestational age was 28.8 (SD 2.6) weeks. There were 539/1511 (35.7%) ELBWI. The majority of the VLBWI was female (822/1507; 54.5%) and most were black (1457/1511; 96.4%). Other neonatal characteristics are shown in Table 1. Caesarean section (CS) was the most common mode of delivery (961/1505; 63.9%). The mean maternal age was 28.7 years (SD 6.1). Other maternal characteristics are shown in Table 2.

**Table 1:** Characteristics of VBLW infants

Characteristic	n/N	Percent
Five minute Apgar <6	221/1490	14.8
Early onset sepsis (<3 days)	62/1423	4.4
Grade 3 or 4 intraventricular haemorrhage	49/774	6.3
Hyperglycaemia	277/1436	19.3
Hypoglycaemia	158/1436	11.0
Metabolic acidosis	93/1436	6.5
Pneumothorax	6/1418	0.4
Respiratory distress syndrome	1260/1430	88.1
Surfactant therapy at any time	1064/1511	70.4
Pulmonary haemorrhage	22/1436	1.5
Nasal CPAP	986/1390	70.9
Mechanical ventilation	231/1359	17.0
Patent ductus arteriosus	125/1419	8.8
Retinopathy of prematurity stage 3 or 4	14/411	3.4
Oxygen on day 28	340/1294	26.3
Steroids for chronic lung disease	195/1299	15.0
Necrotising enterocolitis grade 2 or 3	100/1423	7.0
Late onset sepsis (>3 days)	396/1424	27.8
Cystic Periventricular leukomalacia	10/1019	1.0

**Table 2:** Maternal characteristics

Characteristic	n/N	Percent
Primiparous	448/1448	30.9
Multiple gestation	286/1495	19.1
Mode delivery -%		
Normal vaginal delivery	495/1505	32.9
Vaginal breech	49/1505	3.3
Emergency C Section	891/1505	59.2
Elective C Section	70/1505	4.7
Attended antenatal care	1229/1485	82.8
Antenatal steroids received	749/1425	52.6
Antenatal magnesium sulphate	82/1401	5.4
Chorioamnionitis	49/1444	3.4
Maternal hypertension	435/1446	30.1
Maternal HIV	431/1468	29.4
Maternal syphilis	28/1416	2.0
Teenage mother	34/1483	2.3
Attempted termination of pregnancy	17/1449	1.2

### Outcomes of delivery room resuscitation

Very few VLBWI did not require any DRR (179/1511; 11.8%) and 10.6% (160/1511) received ADRR. The number of VLBWI in each level of DRR is shown in Table 3. Significantly more ELBWI required DRR than bigger infants (83/539 (15.4%) vs 77/972 (7.9%) *p* < 0.001).

**Table 3:** Level of resuscitation in infants <1000 grams at birth vs those > 1000 grams at birth

Level of resuscitation	Total *N* = 1511 n (%)	<1000 grams birth weight *N* = 539 n (%)	>1000 grams birth weight *N* = 972 n (%)	*P* value
Oxygen	1332 (88.2)	507 (94.1)	825 (84.9)	<0.001
Face mask ventilation	683 (45.2)	308 (57.2)	375 (38.6)	<0.001
Intubation	25 (1.7)	11 (2.0)	14 (1.5)	0.381
Adrenaline	22 (1.5)	11 (2.0)	11 (1.1)	0.158
Chest compressions	152 (10.1)	80 (14.8)	72 (7.4)	<0.001

### Outcomes of advanced delivery room resuscitation

The mean birth weight of VLBWI who received ADRR was significantly lower than that of those who did not require ADRR (992.0 grams (SD 255.4) vs 1105.1 grams (SD 256.3) *p* < 0.001). Similarly, the gestational age of VLBWI who required ADRR was significantly less than those who did not (27.9 weeks (SD 2.7) vs 29.0 weeks (SD 2.7) *p* < 0.001). Other maternal and neonatal characteristics that were significantly associated with ADRR are shown in Table 4.

**Table 4:** Maternal and neonatal characteristics significantly associated with the need for advanced delivery room resuscitation in very low birth weight infants

Characteristic	ADRR n/N (%)	No ADRR n/N (%)	*P* value
Antenatal care	120/157 (76.4)	1109/1328 (83.5)	0.026
Antenatal steroids	57/150 (38.0)	692/1275 (54.3)	<0.001
Mode of delivery:			
-Normal vertex delivery	54/159 (34.0)	441/1346 (32.8)	0.008
-Vaginal breech	11/159 (6.9)	38/1346 (2.8)	
-Emergency CS	92/159 (57.9)	799/1346 (59.4)	
-Elective CS	2/159 (1.3)	68/1346 (5.1)	
Five minute Apgar score <6	99/157 (63.1)	122/1333 (9.2)	<0.001
Bacterial sepsis in the first 3 days of life	10/126 (8.9)	52/1297 (4.0)	0.039
Oxygen on day 28	43/107 (40.2)	297/1187 (25.0)	0.001
Hyperglycaemia	34/128 (26.6)	243/1308 (18.6)	0.029
Hypernatraemia	21/128 (16.4)	137/1308 (10.5)	0.041
Metabolic acidosis	20/128 (15.6)	73/1308 (5.6)	<0.001
Respiratory distress syndrome	121/127 (95.3)	1139/1303 (87.4)	0.009
Surfactant therapy	127/160 (79.4)	937/1351 (69.4)	0.009
Died	89/160 (55.6%)	350/1351 (25.9)	<0.001
Died in delivery room	32/160 (20.0)	46/1351 (3.4)	<0.001
Died within 12 hours of admission	41/160 (25.6)	46/1344 (3.4)	<0.001

All other maternal and neonatal characteristics were not significantly associated with ADRR. In particular, admission temperature, NEC, ROP, PDA, IVH, cystic PVL, LOS, pneumothorax, pulmonary haemorrhage and the need for NCPAP or mechanical ventilation were not significantly associated with ADRR. The indication for C section was recorded in 649/961 (67.5%) of VLBWI delivered by C section. There was no significant association between the indication for C section and the need for ADRR (see Table 5). Multivariable regression showed that ADRR remained significantly associated with a 5 minute Apgar score below 6, supplemental oxygen at 28 days of life, metabolic acidosis and death (See Table 6).

**Table 5:** The need for advanced delivery room resuscitation and indication for C section

Indication	No ADRR (582) n (%)	ADRR (67) n (%)	*p* value
Absent end diastolic placental blood flow	7 (1.2)	0 (0)	*p* = 0.243
Antepartum haemorrhage	49 (8.4)	12 (17.9)	
Cord prolapse	2 (0.3)	1 (1.5)	
Decreased liquor	4 (0.7)	0 (0)	
Fetal distress	62 (10.7)	4 (6.0)	
Malpresentation	7 (1.2)	0 (0)	
Miscellaneous	4 (0.7)	1 (1.5)	
Proteinuric hypertension	377 (64.8)	44 (65.0)	
Poor obstetric history	19 (3.3)	1 (1.5)	
Prolonged rupture of membranes	14 (2.4)	1 (1.5)

**Table 6:** Adjusted odds ratios for significant associations with advanced delivery room resuscitation

Characteristic	Odds Ratio	95% CI	*P* Value
Five minute Apgar score <6	13.8	8.6–22.0	<0.001
Supplemental oxygen at day 28	2.2	1.4–3.9	0.001
Metabolic acidosis	2.3	1.1–4.8	0.035
Died	1.9	1.1–3.3	0.027

### Mortality

Overall in- hospital mortality was 29.1% (439/1511); 78/439 (17.8%) of the deaths occurred in the delivery room. There were 87 of the 439 deaths (19.8%) that occurred within 12 hours of admission; the majority of these occurred within the delivery room (65/87; 74.7%). Significantly more ELBWI died compared to those with a birth weight > 1000 grams (322/546; 59.0% vs 127/983; 12.9%) (*p* < 0.001). There was no difference in mortality by gender – 210/685 (30.6%) males died compared to 229/822 (27.8%) females (*p* = 0.255). – The admission temperature of VLBWI who died was significantly lower than those who survived – 35.1 °C (SD 0.92) vs 36.1 °C (SD 1.4). (*p* < 0.001)

**Fig. 1:**
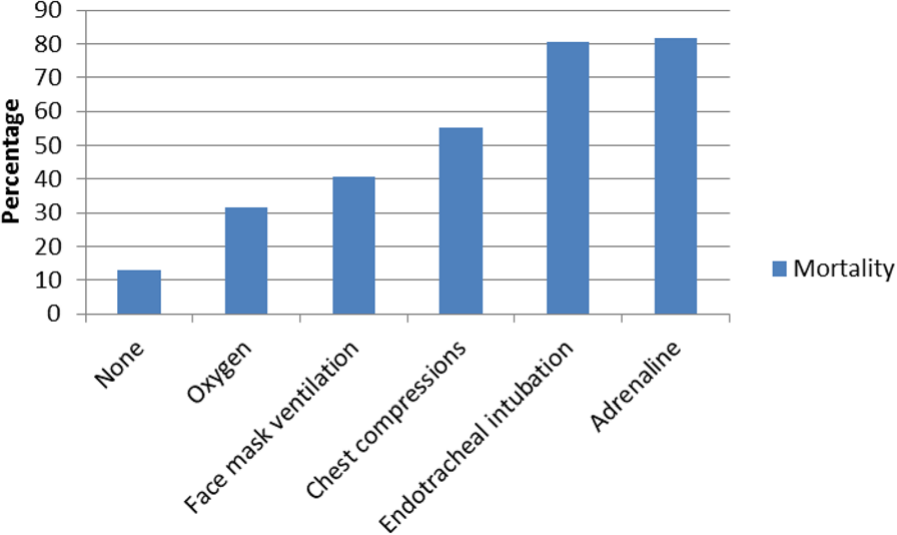
Mortality by level of delivery room resuscitation

Mortality rates by level of resuscitation are shown in Fig. 1. Mortality was significantly associated with advancing levels of DRR (intubation, chest compressions or adrenaline administration) (*p* < 0.001). More than half the VLBWI who required ADRR died (89/160; 55.6%).

Those VLBWI who required ADRR and died within the first 12 hours of admission were compared to those who survived for more than 12 hours. In this group, death within the first 12 hours was significantly associated with lower admission temperature (34.9 °C SD 1.9 vs 36.0 °C SD 1.9; *p* < 0,001), lighter birth weight (865 grams SD 248 vs 1035 grams SD 243; *p* < 0.001) and lower gestational age (26.5 weeks SD 2.7 vs 28.4 weeks SD 2.6; *p* < 0.001).

## Discussion

This review from a tertiary hospital in South Africa shows that almost all VLBWI required some degree of resuscitation in the DR, however only 10.6% needed ADRR. These findings are similar to a report by Cho et al. [8] who found that only 5.9% of VLBWI required chest compressions or drugs. In the current study, both decreasing birth weight and gestational age were significantly associated with the need for ADRR, which is in keeping with other reports [6–10].

Mortality was significantly associated with the need for ADRR in the present study. More than half the VLBWI who needed ADRR died, most of these deaths occurred in ELBWI. Increasing mortality with advancing levels of resuscitation has been reported by other researchers [7–10]. In the current study, most neonatal resuscitation was initiated by junior house staff, senior staff was only called to assist prolonged or difficult resuscitation.Ideally experienced senior staff, including registrars and fellows, should attend all high risk deliveries, including those of VLBWI. It may not be feasible to provide this service in circumstances of limited resources, so adequate training of available staff in neonatal resuscitation should be emphasised.

Although mode of delivery, antenatal care and antenatal steroids were significantly associated with the need for ADRR on univariate analysis, the adjusted odds ratios for these variables were not significant. Similarly, perinatal risk factors, determined by the indication for C-section, were not significantly associated with the need for ADRR. The only labour room characteristic significantly associated with the need for ADRR in the current study was a 5 minute Apgar score below six. Iliodromiti et al. [18] confirmed the usefulness of the Apgar score in modern day practice - both in term and preterm neonates.

Several complications of prematurity were significantly associated with ADRR on univariate analysis, but adjusted odds ratios showed that only metabolic acidosis and the need for supplemental oxygen at 28 days of life remained significant. A review of VLBWI who were entered into the Caffeine and Apnoea of Prematurity (CAP) trial showed that BPD was significantly associated with ADRR [6].

Admission hypothermia significantly increases the chance of death in very preterm infants [19, 20]. In the present study, admission temperature was significantly lower in the VLBWI who died. Of particular note, VLBWI infants who required ADRR and died within the first 12 hours of life were significantly smaller, more preterm and had a lower admission temperature. This highlights the importance of preventing hypothermia in the smallest VLBWI. Although polythene wrap, radiant warmers and a warm delivery room are routine practice in the study centre, more care needs to be taken to ensure extreme preterm infants do not become hypothermic. Other measures to prevent neonatal hypothermia should be implemented, such as use of caps, avoiding cold air for resuscitation, reducing maternal hypothermia and ensuring normothermia during transfer between wards [19].

In the present study, no major complications of prematurity, including ROP, NEC, IVH, cystic PVL, sepsis, pneumothorax, pulmonary haemorrhage or the need for ventilatory support were significantly associated with ADRR. This is in contrast to many other reports which have reported an increase in complications of prematurity in VLBWI who had ADRR [6–8]. This finding may be related to the high mortality rate associated with ADRR in the present study – more than half the VLBWI needing ADRR died. Another possible reason relates to the definition of ADRR in the present study which included the need for intubation. Very few VLBWI (1.7%) were actually intubated, in strong contrast to a report from Korea where 77.7% of VLBWI were intubated at birth [8]. In addition, in the present study far more VLBWI received chest compressions than were intubated. This reflects the neonatal resuscitation training according to the South African Neonatal Resuscitation Algorithm (SANRA) (http://www.resus.co.za). Most birth attendants are midwives, so the training emphasises effective face mask ventilation, rather than intubation. In the SANRA, health workers who are not skilled in intubation are taught to intubate after chest compressions. Those VBLWI with RDS at birth would not be routinely intubated as NCPAP was the first line ventilatory support in the unit at the time of the study.

Complications of prematurity were not increased in survivors of VLBWI who required ADRR. This suggests that survivors of ADRR should be provided with ventilatory support if required– the need for ADRR alone is not sufficient reason to ration care. Some other researchers have reported that the outcome of VLBWI following ADRR is not that bad. In a study from the Vermont Oxford Network in the late 1990s Finer et al. reported that most VLBWI undergoing ADRR survive and half did not have severe IVH [7]. De Mauro et al. reported on the cohort of VLBWI enrolled in the CAP trial and found that ADRR was not associated with worse neurodevelopmental outcome at 18 months [6]. Other factors apart from the need for ADRR should be considered when making decisions to withhold or withdraw care in these VLBWI.

There was a high mortality rate in VLBWI requiring ADRR in a tertiary centre with neonatal facilities. Although there is no available information, it is very probable that the mortality rate of VLBWI is even higher in non-tertiary centres where DRR may be sub-optimal. This emphasises the need for VLBWI to be delivered in a properly equipped tertiary centre, where possible. In addition, available staff in non-tertiary centres should be properly trained and equipped to carry out neonatal resuscitation.

### Limitations of the study

The retrospective nature of the study meant that some data was missing and could not be evaluated. More detailed information regarding the resuscitation e.g. dose of adrenaline, duration of chest compressions, number of attempts at intubation could not be evaluated. There was no record of the timing of cord clamping, so the effect of delayed cord clamping could not be determined. The benefit or otherwise of the seniority and experience of the attending staff members conducting the resuscitation could not be assessed. The number of VLBWI who had cranial sonars done was low due to resource constraints, so the incidence of IVH and PVL may be under estimated. Rates of death and complications are a poor proxy for the quality of resuscitation. A prospective study should be done to appraise the quality and effectiveness of DRR in VLBWI.

## Conclusion

The present study showed that mortality increased with advancing levels of delivery room resuscitation in VLBWI. Extremely preterm, small VLBWI were most likely to die after ADRR. Major complications of prematurity were not increased in those VLBWI who survived ADRR. All VLBWI who survive ADRR should be provided with ventilatory support if required; the results of the present study suggest that the need for ADRR alone is not sufficient reason to limit care in these infants in the study setting. The low complication rate, however, may be related to high mortality in this group of infants – improved survival may result in an increase in complications of prematurity. There must be careful monitoring of the outcome of VLBWI who are provided with ventilatory support.

## Abbreviations

ADRR: Advanced delivery room resuscitation
BPD: Bronchopulmonary dysplasia
CAP: Caffeine and apnoea of prematurity
CMJAH: Charlotte Maxeke Johannesburg Academic Hospital
CS: Caesarean section
DRR: Delivery room resuscitation
ELBWI: Extremely low birthweight infants
EOS: Early onset sepsis
IVH: Intraventricular haemorrhage
LOS: Late onset sepsis
NCPAP: Nasal continuous positive airways pressure
NEC: Necrotising enterocolitis
NICU: Neonatal intensive care unit
PDA: Patent ductus arteriosus
PVL: Periventricular leukomalacia
RDS: Respiratory distress syndrome
REDCAP: Research electronic data capture
ROP: Retinopathy of prematurity
SRT: Surfactant replacement therapy
VLBWI: Very low birthweight infants
